# Metal Artefact Reduction Sequences (MARS) in Magnetic Resonance Imaging (MRI) after Total Hip Arthroplasty (THA): A non-invasive approach for preoperative differentiation between periprosthetic joint infection (PJI) and aseptic complications?

**DOI:** 10.1186/s12891-022-05560-x

**Published:** 2022-06-28

**Authors:** André Busch, Marcus Jäger, Sascha Beck, Alexander Wegner, Erik Portegys, Dennis Wassenaar, Jens Theysohn, Johannes Haubold

**Affiliations:** 1 Department of Orthopaedics and Trauma Surgery, Philippusstift Essen, Hülsmannstr. 17, 45355 Essen, Germany; 2 Chair of Orthopaedics and Trauma Surgery University of Duisburg-Essen, Essen, Germany; 3 Department of Orthopaedics and Trauma Surgery, Sportklinik Hellersen, Paulmannshöher Str. 17, 58515 Lüdenscheid, Germany; 4 Department of Orthopaedics, Trauma and Reconstructive Surgery, Marienhospital Mülheim an Der Ruhr, Kaiserstraße 50, 45468 Mülheim, Germany; 5 Institute of Diagnostic and Interventional Radiology and Neuroradiology, University Hospital Essen, Hufelandstrasse 55, 45147 Essen, Germany

**Keywords:** MARS, THA, MRI, periprosthetic joint infection, Synovial layering

## Abstract

**Background:**

In the past, radiographic imaging was of minor relevance in the diagnosis of periprosthetic joint infections (PJI). Since metal artefact reduction sequences (MARS) are available, magnetic resonance imaging (MRI) has become a promising diagnostic tool for the evaluation of hip arthroplasty implants. The purpose of the present study was to evaluate the efficacy of MARS-MRI in comparison to established diagnostic tools to distinguish between aseptic failure and PJI.

**Methods:**

From July 2018 to September 2019, 33 patients classified as having an aseptic joint effusion were recruited into the study. The group included 22 women and 11 men with a mean age of 70.4 ± 13.7 (42–88) years. In the same period, 12 patients were classified as having a PJI. The group consisted of 9 women and 3 men with a mean age of 72.5 ± 10.6 (54–88) years. MARS-MRI was conducted using the optimized parameters at 1.5 T in a coronal and axial STIR (short-tau-inversion recovery), a non-fat-saturated T2 in coronal view and a non-fat-saturated T1 in transverse view in 45 patients with painful hip after total hip arthroplasty (THA). Normally distributed continuous data were shown as mean ± standard deviation (SD) and compared using student's t-test. Non-normally distributed continuous data were shown as mean and compared using the Mann–Whitney U test.

**Results:**

Synovial layering and muscle edema were significant features of periprosthetic joint infection, with sensitivities of 100% and specifities of 63.0—75.0%. The combined specifity and sensitivity levels of synovial layering and muscular edema was 88.0% and 90.0%. Granulomatous synovitis was a significant feature for aseptic failure, with 90.0% sensitivity and 57.0% specifity.

**Conclusion:**

MARS-MRI is as suitable as standard diagnostic tools to distinguish between aseptic failure and PJI in patients with THA. Further studies with larger patient numbers have to prove whether MARS-MRI could be integral part of PJI diagnostic.

## Background

The main reason for prosthetic failure in total joint arthroplasty (TJA) is aseptic loosening [1]. Other reasons for prosthetic failure are recurrent dislocations (hip) and periprosthetic joint infections (PJI) [2]. The five-year incidence of periprosthetic hip joint infections exceeds one percent following primary procedure [3]. The differentiation between aseptic failure and PJI is of central importance because the treatment of aseptic failure is completely different from the treatment of PJI [4, 5]. Serum and synovial biomarkers (CRP, Alpha-1-Defensin) are frequently used to diagnose PJI [6–8]. However, there are cases in which serum and synovial biomarkers fail [9]. In the past, radiographic tools were of minor importance in ruling out PJI. Conventional X-ray and scintigraphic imaging is not sufficient to differentiate between aseptic processes and PJI [10–12]. Cross-sectional imaging had the disadvantage that metal-induced artefacts result in impaired image quality [13]. Due to technical innovations such as MARS (metal artefact reduction sequences), it is now possible to assess periprosthetic soft tissue and bone changes in magnetic resonance imaging (MRI) [14, 15]. First reports on radiographic imaging via MARS-MRI identified characteristic features such as granulomatous synovitis and synovial layering to differentiate painful arthroplasty [15, 16]. However, until now no study compared the diagnostic value of MARS-MRI in painful hip arthroplasty with those of standard diagnostic tools.

The purpose of the current study was to evaluate the accuracy of MARS-MRI in distinguishing between aseptic failure and PJI in painful hip after total hip arthroplasty (THA). The hypothesis to be tested was that MARS-MRI is suitable to differentiate non-invasively between PJI and aseptic complications in THA as valuable as serum and synovial biomarkers do.

## Methods

### Study design

After approval from the institutional review board (18–8042-BO), a prospective study was performed in patients with persisting pain [17] after THA. Medical history, clinical examinations, and laboratory values of serum and joint fluid were gathered preoperatively as routine diagnostic procedures. In all patients the intraoperative microbial test were available. There were no differences between the pre- and intraoperative microbial test results. The differentiation between aseptic failure and PJI was made according to the 2018 definition of periprosthetic hip and knee infection [18] (Table 1). The criterion for inclusion was persisting pain after THA [17]. Patients were excluded if primary implantation or any surgery on the affected hip had been performed within the last 12 months. Further reasons for exclusion were contraindications for MRI such as cardiac pacemakers.

**Table 1:**
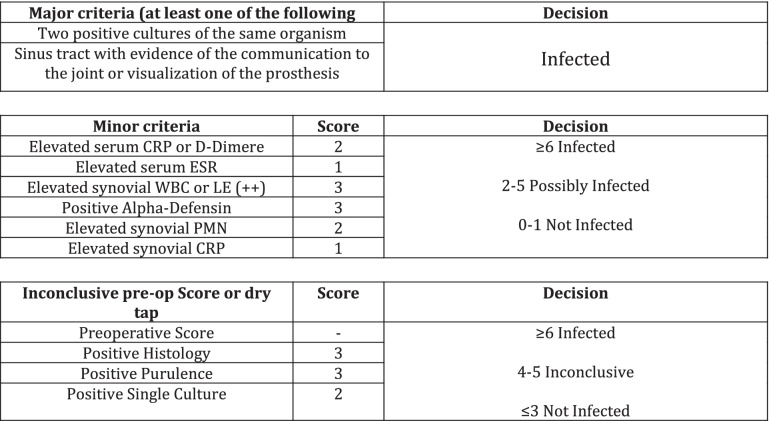
2018 Definition of periprosthetic hip and knee infection

### Ethics statement

The study was approved by the ethics committee of the investigating hospital. Written informed consent was obtained from all individual participants included in the study.

### Patients

From July 2018 to September 2019, 33 patients classified as having an aseptic joint effusion were recruited into the study. The group included 22 women and 11 men with a mean age of 70.4 ± 13.7 (42–88) years. The mean BMI was 28.6 ± 5.4 (21–40). In the same period, 12 patients were classified as having a PJI. The group consisted of 9 women and 3 men with a mean age of 72.5 ± 10.6 (54–88) years. The mean BMI was 29.2 ± 5.0 (21–37). Bacteria were identified in 10 (83%) of 12 patients of the infection group. Staphylococci were found in 6 (60%) and Pseudomonas, Serratiae, Klebsiellae and Lactobacillae were found in each one (10%). In 2 patients (17%) in the infection group, no bacteria could be isolated after 14 days incubation. There were no significant differences in sex, age, and BMI between the cohorts (*p* > *0.05*). In all patients, revision surgery was performed due to failure of primary implant. None of the patients suffered from inflammatory joint disesase. Twenty-two patients (15 aseptic and 7 PJI) had ceramic-polyethylene and 23 (17 aseptic and 5 PJI) metal-polyethylene load bearing. In seven patients (6 metal and 1 ceramic head) MRI analysis was not possible due to persisting interfering artefacts.

Determination of the Levels of Serum and Synovial Fluid biomarkers: Serum CRP was analyzed by immune turbidimetry (Centaur, Siemens, Germany, normal < 0.5 mg/dl). Synovial leukocyte level and percentage of polymorphic neutrophils were measured by flow cytometry with EDTA (Ethylenediaminetetraacetic acid) plasma (XN 550, Sysmex, Germany) (normal < 3000/μl and < 80%). Synovial biomarkers were analyzed using standard quantitative enzyme immunoassay kits (1. Human α-Defensin 1 Antibody, R&D Systems Bio-Techne, USA, cutoff level 4800 ng/mL; 2. CRP (EU59131), IBL International GmbH, Hamburg, Germany / cut-off level (> 6,9 mg / l)).

### MR-Imaging

A 1.5 T Philips Intera (Koninklijke Philips N.V., Amsterdam, Netherlands) MR scanner was used to acquire MR images using a Philips SENSE XL Torso 16-channel coil. Standardized imaging protocols were used containing a short tau inversion recovery sequence (STIR) in coronal (repetition time (TR) 3130 ms, echo time (TE) 35 ms, in plane resolution 0.8 × 0.8 mm, slice thickness 3.5 mm) and transverse view (TR 4428 ms, TE 40 ms, in plane resolution 0.9 × 0.9 mm, slice thickness 3.5 mm), as well as a non-fat-saturated T2 in coronal view (TR 3076 ms, TE 80 ms, in plane resolution 0.7 × 0.7 mm, slice thickness 2.5 mm) and a non-fat-saturated T1 in transverse view (TR 669 ms, TE 10 ms, in plane resolution 0.7 × 0.7 mm, slice thickness 2.5 mm). To reduce metal artefacts all sequences were acquired using Philips MARS technique. The time between MRI and revision surgery was 4.8 days (1–9).

### MRI criteria

The MRI features used to distinguish between PJI and aseptic complications included edema of the synovium, bone edema, muscle edema, fistulae between the joint space and the surrounding soft tissue, bursitis signs, granulomatous synovitis and lamellation (layering) of the synovium [16, 19–21].

Consistency test: The investigation of the MRI images was performed by two authors (JH, AB). Cases were rereviewed 2 weeks later by each reader. Both readers were not involved in patients recruitment and data collection.

### Statistical analysis

The data were processed with the statistical software package SPSS. Normally distributed continuous data were shown as mean ± standard deviation (SD) and compared using student's t-test. Non-normally distributed continuous data were shown as mean and compared using the Mann–Whitney U test. A p-value < 0.05 was considered statistically significant. Sensitivity and specificity for any cut-off level were calculated. ROC curves were subsequently constructed by mapping true-positive rate (sensitivity) against false-positive rate (1 − specificity) for each test-joint combination. Inter- and intrareader agreement of imaging findings was assessed with Fleiss’ κ for binary parameters.

## Results

The appearance of synovial layering in MARS-MRI was significantly higher in PJI than in aseptic complications (*p* < *0.05*). Synovial layering demonstrated a sensitivity of 100% and a specificity of 73% for the diagnosis of PJI in this study. Muscle edema were not significantly higher in PJI than in aseptic complications (*p* = *0.11*). Muscle edema had a specifity and a sensitivity of 65% and 100%, respectively, for the diagnosis of PJI. When synovial layering was combined with muscular edema, the levels of specificity and sensitivity were 88% and 90%, respectively, for the diagnosis of PJI in this study. Granulomatous synovitis was significantly higher in aseptic complications than in PJI (*p* = *0.03*). Granulomatous synovitis had a sensitivity of 90% and a specifity of 57% for the diagnosis of aseptic failure. Acetabular and femoral bone marrow edema were not significantly higher in PJI than in aseptic cohort (*p* = *0.27* and *p* = *0.11*). Fistulae between the joint space and the surrounding soft tissue and bursitis trochanterica were not significantly higher in PJI than in aseptic complications (*p* = *0.64* and *p* = *0.72*) (see Table 2a).

**Table 2:**
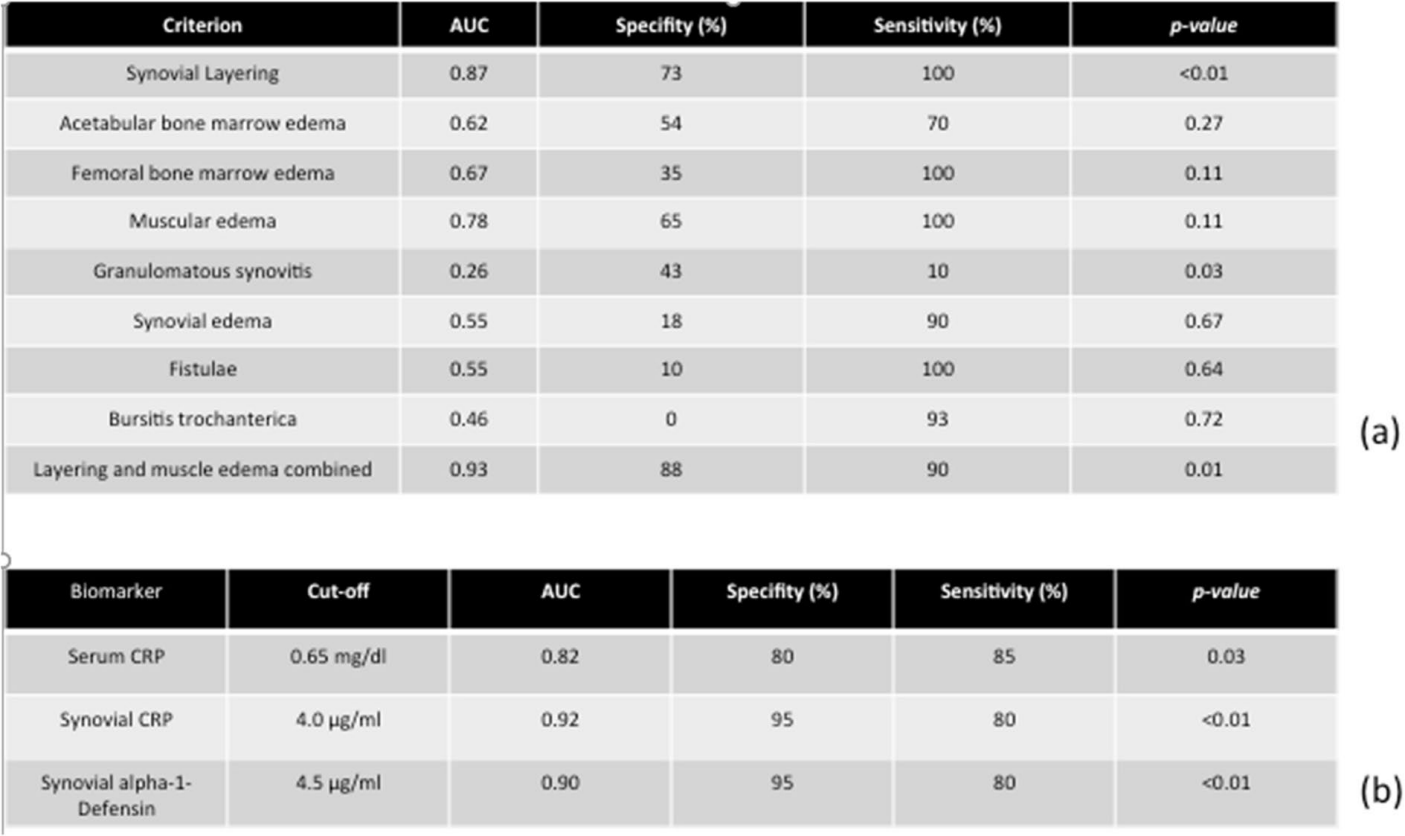
(a) Evaluation of MRI criteria for the diagnosis of periprosthetic joint infection. (b) Evaluation of serum and synovial fluid biomarkers for the diagnosis of periprosthetic joint infection

The mean levels of synovial alpha-1-Defensin were significantly higher in PJI than in aseptic complications (*p* < *0.01*). Synovial alpha-1-defensin showed a sensitivity of 80% and a specifity of 95% with a cut-off of 4,8 μg/ml. The average levels of synovial (*p* < *0.01*) and serum (*p* = *0.03*) CRP were significantly higher in PJI than in aseptic complications. Serum CRP had a sensitivity of 85% and a specifity of 80%. Synovial CRP showed a sensitivity of 80% and a specifity of 95% (see Table 2b).

Figures 1, 2, 3 and 4 show radiographic images from a patient suffering from PJI. Figures 5 and 6 demonstrate the x-ray and MR images from a patient with aseptic complication after THA. Figure 7 displays the ROC plot of the MRI criteria. Figure 8 shows the ROC plot of serum and synovial biomarkers.

**Fig. 1:**
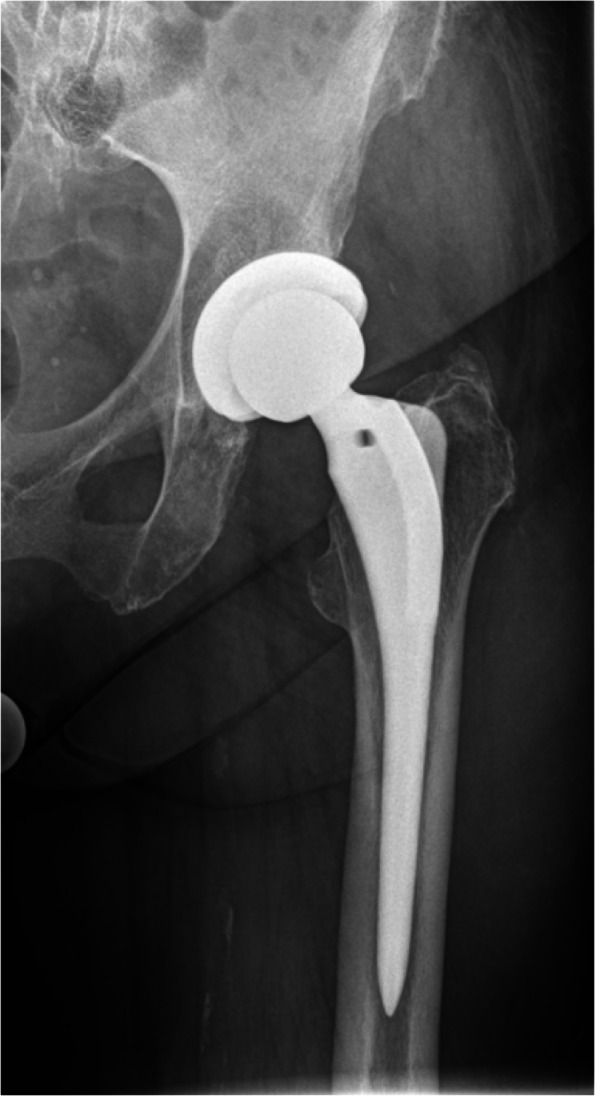
X-ray from a patient suffering from PJI. Plain radiograph showing normal findings

**Fig. 2:**
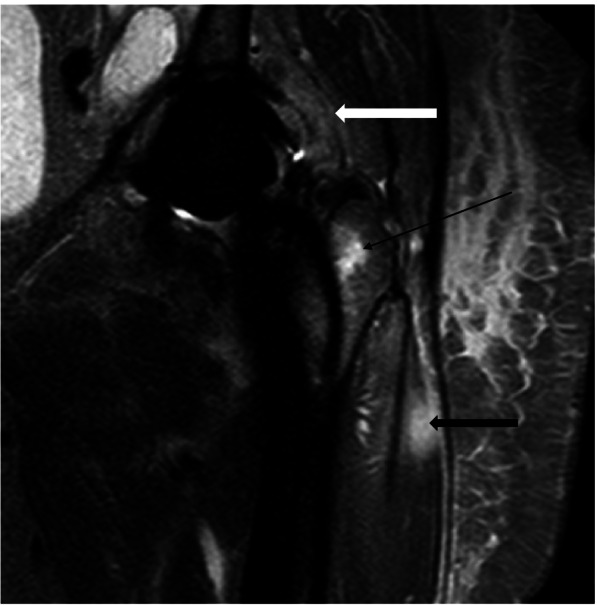
Coronal MARS-STIR-MRI shows layering (white arrow) and hyperintensity of the synovium which is highly suggestive of an infectious process. Femoral bone marrow (black thin arrow) and muscle edema (black thick arrow) indicates periprosthetic stress reaction

**Fig. 3:**
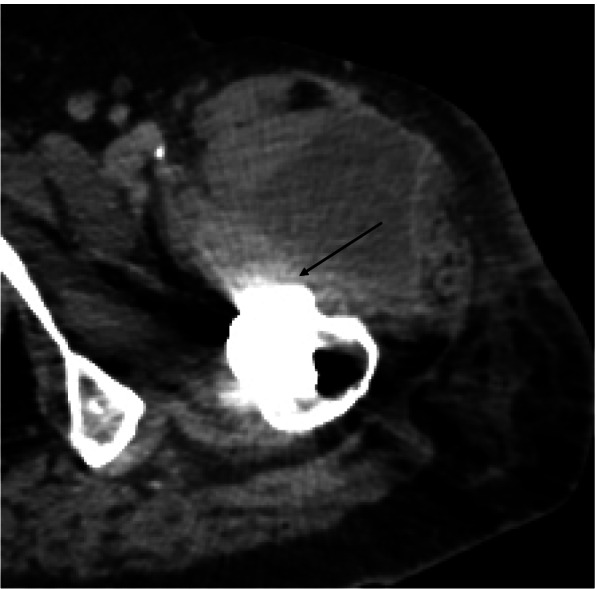
Axial CT shows field accumulation anterior to femoral bone. Due to beam hardening (black arrow), the soft tissue surrounding the periprosthetic bone is not evaluable

**Fig. 4:**
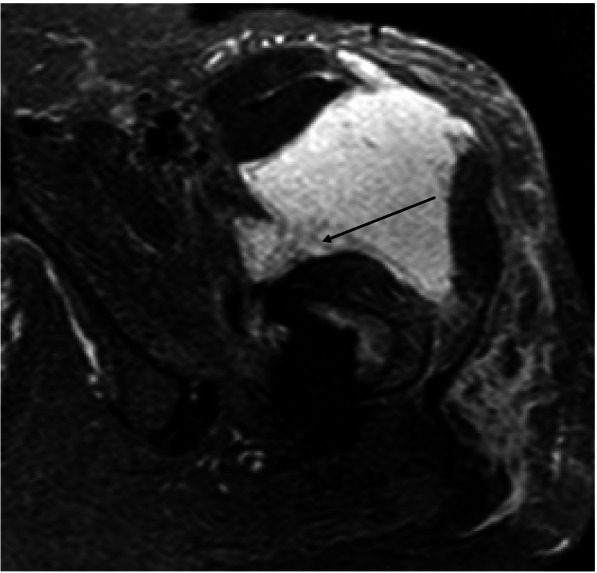
On axial MARS-STIR-MRI layering (black arrow) is detectable in the fluid accumulation

**Fig. 5:**
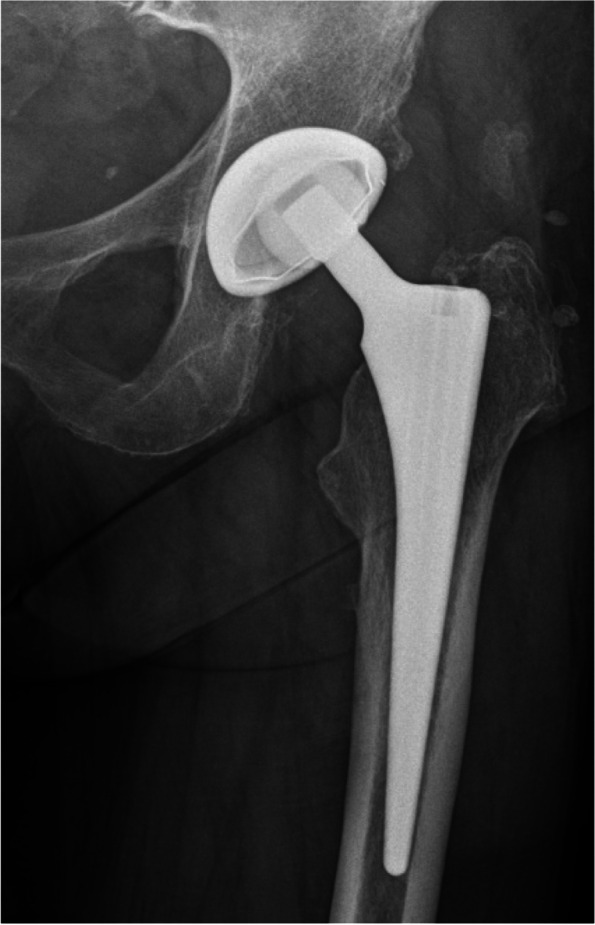
Plain radiograph shows asymmetric polyethylene wear of the inlay

**Fig. 6:**
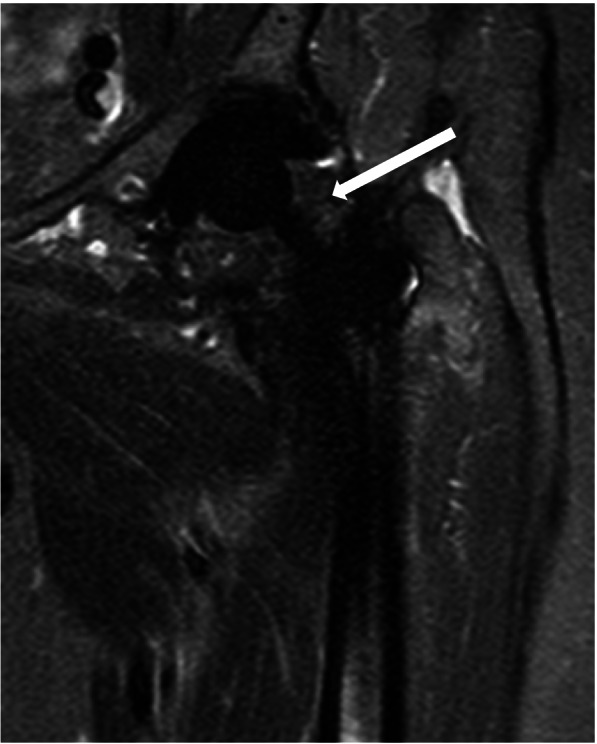
On coronal MARS-STIR-MRI granulomatous synovitis (white arrow) indicates polyethylene wear debris

**Fig. 7:**
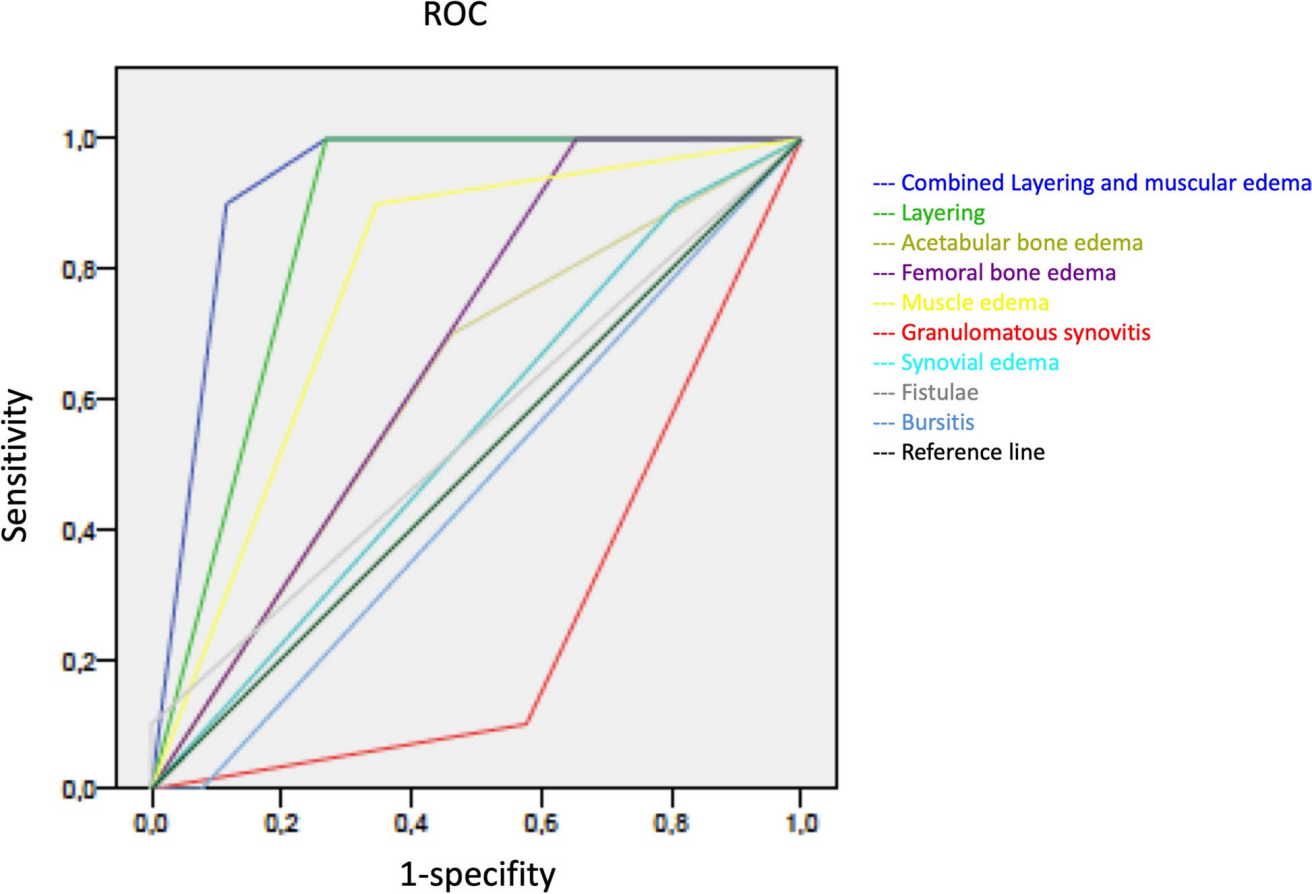
ROC plot for edema of the synovium, bone edema, muscle edema, sinus tracts, bursitis signs, granulomatous synovitis and layering of the thickened hyperintense synovium

**Fig. 8:**
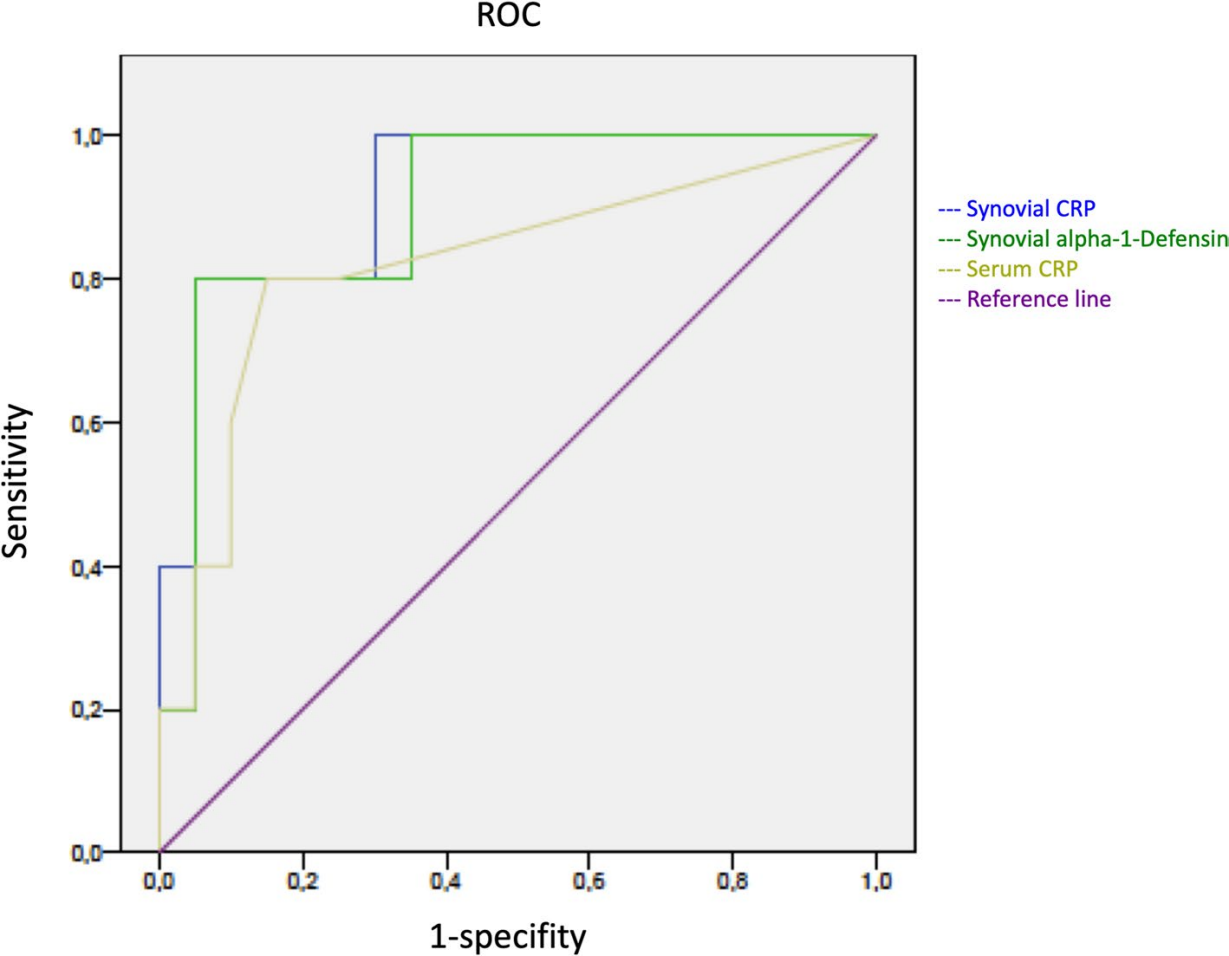
ROC plot for serum CRP, synovial alpha-1-defensin and CRP

## Discussion

The diagnosis of PJI is still a major problem in orthopaedic surgery because so far no “gold-standard” has been established [22]. Radiographic imaging is a keystone in orthopaedic diagnostics because it is non-invasive and available almost everywhere. However, until recently radiographic imaging was not considered to be suitable to rule out PJI [23]. Plain radiographs of septic hip prostheses often show normal findings but can exhibit periosteal new bone formation and scattered foci of osteolysis during the subsequent course [24, 25]. Cross-sectional imaging in post-arthroplasty hips has always been a challenge because of the susceptibility artefacts on MRI and beam hardening on computed tomography (CT) [25]. For those reasons, various mitigation strategies have been developed to minimize these artefacts [26–30]. On the one hand, it has been shown that these artefacts increase with a field strength above 1.5 T [31]. Therefore, the patients in this study were examined at 1.5 T. Determining whether our results can be transferred to higher field strengths will require further investigations. On the other hand, our results were obtained exclusively with the Philips MARS technique; however, overall, several manufacturers have established similar techniques with well-proven capabilities for postoperative metal artefact reduction [32–34]. In our study setting, we were not able to verify transferability to other sequences from other manufacturers; however, the current study landscape suggests that our results can be obtained with similar sequencing techniques from other manufacturers [35–37].

Fritz et al*.* (2014) were one of the first to describe MRI features in post-arthroplasty hips. Suggestive features for PJI were sinus tracts, abscess formation, bone marrow edema and lymphadenopathy [15]. No patient in our study suffered from a sinus tract with the evidence of the communication to the joint or the visualization of the prosthesis.

The MRI features in our study included (a) synovial layering; (b) edema of femoral and acetabular bone marrow; (c) muscular edema; (d) granulomatous synovitis; (e) synovial edema; (f) fistulae between the joint space and the surrounding soft tissue; and (g) bursitis trochanterica. Data analysis revealed significant differences between the two cohorts in two parameters. Synovial layering was significantly more frequent (*p* = *0.01*) in patients with PJI than in aseptic complication. Synovial layering was defined as a thickened synovium composed of multiple lamelles. Plodowski et al.firstly described the presence of synovial layering at MR imaging of knee arthroplasty. They found that synovial layering had a high sensitivity and specificity for detection of PJI [19]. According to Plodowski et al., we found synovial layering to have excellent sensitivity (100%) to diagnose PJI. The specifity of synovial layering with 73% is, however, limited. To increase specifity in diagnosis of PJI the combination of synovial layering and edema in the surrounding muscle tissue can be applied (specifity of synovial layering and muscle edema combined = 88%). However, the sensitivity of combined synovial layering and muscle edema is below the sensitivity of synovial layering alone (90% vs. 100%).

In TJA polyethylene debris causes synovitis, cytokine driven up-regulation of osteoclasts and down regulation of osteoblasts resulting in osteolysis with consecutive implant loosening [38]. Many efforts have been made to improve durability of polyethylene implants in TJA. By introduction of cross-linked and Vitamin-E supplemented polyethylene implants the wear rates could be decreased. However, polyethylene wear debris induced periprosthetic osteolysis remains one of the main causes for aseptic loosening in THA [39]. MARS-MRI was described to be an appropriate modality to detect polyethylene wear debris induced synovitis [15]. Polyethylene wear–induced synovitis was characterized to occur as an expansion of the hip pseudocapsule by a thick and particulate appearing synovitis of low to intermediate signal intensity in MRI [40]. In our study, granulomatous synovitis was significant more often (*p* = *0.03*) in aseptic complications than in PJI. With a sensitivity of 10%, granulomatous synovitis is not suitable to detect PJI. Conversely, granulomatous synovitis seems to be appropriate to diagnose aseptic complication. The intermediate specifity (43%) of granulomatous synovitis to detect PJI might result from an additional occurrence of aseptic soft-tissue reaction due to wear debris.

To our best knowledge, this is one of the first studies reporting on clinical, laboratory and MRI data of patients suffering from painful hip after THA [16]. Our findings indicate that MARS-MRI is a suitable modality for the evaluation of soft and bone tissue adjacent to hip arthroplasty implants. The occurrence of synovial layering and muscle edema combined significantly differed between PJI and aseptic complications (*p* = *0.01*). The accuracy of MARS- MRI parameters were comparable with those of standard serum and synovial biomarkers. Synovial layering and muscle edema combined had the highest AUC level (0.93), even higher than those of synovial CRP (0.92) and synovial AD-1 (0.90) (see Table 2). MARS-MRI may improve the planning of revision arthroplasty in ambiguous cases. The risk of overlooked PJI with the need of further surgery might be reduced.

Previous studies by Fritz et al. (2014) and Jiang et al. (2016) investigating MRI strategies for hip arthroplasty implants have reported similar results [15, 41].

Schwaiger et al. (2020) also found MRI with metal artefact reduction to be accurate to differentiate between patients PJI and aseptic loosening. In contrast to our study, Schwaiger et al. (2020) additionally evaluated patients without aseptic implant loosening and without PJI as a reference group. They found STIR signal hyperintensity in surrounding acetabular and femoral bone marrow indicating edema significantly higher in PJI and aseptic loosening than in the reference group (p < 0.001). The specifity and sensitivity of acetabular bone marrow edema was described to be 88% and 63%, respectively. The specifity and sensitivity of femoral bone marrow edema was found to be 68% and 91%, respectively [42]. These data also argue in favour of using MARS MRI in diagnostic of painful hip after THA because the pathological findings in PJI and aseptic loosening are statistically less frequent in patients without aseptic implant loosening and without PJI.

Galley et al. (2020) also determined the diagnostic performance of 1.5-T MRI with metal artefact reduction to detect PJI after THA [43]. From the 140 patients in their study 40 suffered from PJI. The control group included patients undergoing MRI at least six weeks after THA, with the absence of trauma and no signs for infection. They found periosteal reaction, capsule edema and intramuscular edema to be significantly higher in patients with PJI. In accordance with our study, intramuscular muscular edema showed high sensitivity (95%) and specifity (86%) to detect PJI. Another accordance of both studies were the high rates of sensitivity of capsule (synovial) edema (our study 90%, Galley 83%). However, we found poor specifity (18%) while Galley et al. (2020) found excellent specifity of capsule edema (95%) to detect PJI. This is maybe due to the fact that our control group consisted of patients with aseptic complications (e.g. asymmetric inlay wear) and the need to undergo revision arthroplasty. In the control group of Galley et al. (2020) more than half of the patients suffered from musculotendinous complications (52%). To our best knowledge, there are less studies reporting on musculotendinopathy after THA and the information about synovial inflammation is lacking [44, 45]. An interesting observation described by Galley et al. (2020) is the significantly higher presence of periostal reaction in PJI [43]. Periostal reaction in x-ray was also described to be a clue to the presence of infection [46].

Another interesting feature in MRI after THA is the presence of locoregional lymphadenopathy which was described by Albano et al. (2020) [47]. Inguinal, obturator and iliac lymph nodes of the affected hip were assessed and normalized to those of the unaffected hip to calculate the ratio of nodal size (RNS), ratio of node number (RNN), difference of nodal size (DNS), and difference of node number (DNN). The accuracy of nodal indices ranging from 84.8% (RNS) and 93.1% (RNN).

The current study has limitations. Firstly, in 15% (7/45) the metal artefact reduction was insufficient to allow the MR images to be evaluated. Therefore, further research to decrease the occurrence metal artefacts needs to be performed. Standard protocols with defined MRI features have to be implemented. We are also aware that full conformity of pre- and intraoperative microbial results is rare. This may have been due to the small size number and high accuracy of microbiological analysis. Furthermore, the small number of patients does not allow statistical conclusions.

Another problem in research on PJI is the lack of an internationally recognized definition for PJI. At present, there are no tests that can definitively exclude infection [43]. This requires a combination of tests with high statistical power allowing the clinician to rule out or to confirm PJI. Over the years, a variety of attempts was conducted to define criteria for diagnosing PJI [48]. The evidence-based 2018 definition of periprosthetic hip and knee infection which we used to distinguish between PJI and aseptic complications is widely adopted and was demonstrated to have higher sensitivity and equal specificity compared to existing criteria [18, 49, 50]. However, the definition was supported by only 68% of delegates at the reconvened ICM (International Consensus Meeting on Musculoskeletal Infection) in 2018 and also not endorsed by MSIS (Musculoskeletal Infection Society) [48, 50]. The significant rates of false-positive and false negative test were repeatedly criticized. Even combining tests did not resolve this problem [49]. Furthermore, the diagnostic performance of the 2018 definition was found to better in hip than in knee [50]. Recent research and committee work led to the publication of the EBJIS definition of periprosthetic joint infection in 2019 [48]. It was stated that it was not practical to have a binary definition; infected or not infected. Therefore, a three-level definition is proposed [48].

1. Infection unlikely
2. Infection likely
3. Infection confirmed

It is crucial to note that the significance of each test is different in each group. The group “Infection likely” representing the overlap between aseptic and septic cases should alert the clinician that an infection is not ruled out and further comprehensive investigations are needed. However, multiple positive suggestive tests in this group do not confirm infection. This requires an identification of a positive test from the confirmatory criteria [48].

We were able to confirm the hypothesis that MRI is suitable to differentiate non-invasively between PJI and aseptic complications in THA without using contrast agents, radioactive tracers. Our data suggest that MARS-MRI is as valuable as determination of serum and synovial biomarkers to differentiate between septic and aseptic complication in TJA.

## Conclusion

MARS-MRI appears to be a reliable method of distinguishing between aseptic complications and PJI. The combination of synovial layering and surrounding muscle edema seems to be an indicator for PJI. Further research with standardized protocols and higher patient numbers has to be performed to investigate the role of MARS-MRI in the diagnosis of PJI.

## Data Availability

All patient-related data were collected by file research from the archives the Department of Orthopaedics and Trauma Surgery of the University of Duisburg-Essen/Germany. The raw data can be requested at: a.busch@kk-essen.de.

## Abbreviations

PJI: Periprosthetic joint infection
MARS: Metal artefact reduction sequences
MRI: Magnetic resonance imaging
STIR: Short-tau-inversion recovery
TJA: Total joint arthroplasty
THA: Total hip arthroplasty
EDTA: Ethylenediaminetetraacetic acid
TR: Repetition time
SD: Standard deviation
CT: Computertomography
AD-1: Alpha-1-Defensin
CRP: C-reactive proteine
